# Artificial intelligence in early heart failure detection: from algorithms to bedside integration

**DOI:** 10.3389/fcvm.2026.1717911

**Published:** 2026-03-30

**Authors:** Esteban Zavaleta-Monestel, Sebastián Arguedas-Chacón, Mario Osvaldo Speranza-Sánchez

**Affiliations:** 1 Health Research Department, Hospital Clínica Bíblica, San José, Costa Rica; 2 Cardiology Department, Hospital Clínica Bíblica, San José, Costa Rica

**Keywords:** AI-enabled stethoscopes, AI-enhanced ECG, artificial intelligence, heart failure, point-of-care systems

## Introduction

Heart failure (HF) affects more than 64 million individuals worldwide (1) and remains a leading cause of morbidity, hospitalization, and premature mortality. Its prevalence continues to rise, driven by population aging, improved survival after myocardial infarction, and the growing burden of hypertension and diabetes mellitus (2, 3). HF, therefore, represents a significant public health challenge, with high rates of rehospitalization and escalating healthcare costs totaling billions of dollars each year (4).

A central obstacle in HF management is delayed diagnosis. Up to half of patients receive their initial diagnosis only upon hospitalization for decompensated HF, at which point structural cardiac damage is often advanced and prognosis significantly worse (2, 3). This late recognition reduces the opportunity to initiate guideline-directed medical therapy promptly. Underdiagnosis is also common, as asymptomatic left ventricular systolic dysfunction frequently remains undetected until symptoms become evident (5).

Although echocardiography is the diagnostic gold standard, it is resource-intensive, requires trained personnel, and is not consistently available in primary care or resource-limited settings. As a result, many patients experience delays between symptom onset, referral, and confirmatory imaging. This diagnostic gap highlights the urgent need for scalable, low-cost, and accessible tools that enable earlier detection (6, 7).

Artificial intelligence (AI) offers a promising opportunity to improve this paradigm. By analyzing electrocardiograms (ECGs), digital stethoscope recordings, and electronic health record (EHR) data, AI systems can detect risk states or subtle physiological signatures before they become clinically apparent (8, 9). Between 2023 and 2025, the field advanced rapidly, supported by prospective and randomized studies and cost-effectiveness analyses (8, 10, 11).

This review summarizes recent progress in three key domains: AI-enhanced ECG, AI-enabled stethoscopes, and system-level integration with economic considerations. It outlines the opportunities and challenges associated with their responsible adoption. Unlike prior reviews, this manuscript integrates evidence from AI-enhanced ECG, AI-enabled stethoscopes, and system-level economic and implementation frameworks, highlighting their combined potential to shift the HF diagnostic pathway. This multidomain perspective provides a more comprehensive evaluation of early detection strategies than previous publications.

### AI-Enhanced ECG: prediction and prognosis

The electrocardiogram (ECG) is one of the most widely used diagnostic tests in medicine, and it is low-cost, non-invasive, and accessible even in primary care or resource-constrained settings (12). For decades, its role in HF diagnosis was limited, but artificial intelligence has substantially expanded its diagnostic and predictive capabilities (13).

Attia et al. first demonstrated that AI applied to 12-lead ECGs could accurately identify reduced left ventricular ejection fraction (14). Building on this work, Dhingra et al. showed that AI-enhanced ECG could stratify the risk of incident HF across diverse populations, including the UK Biobank and the Estudo Longitudinal de Saúde do Adulto (ELSA) Brasil cohorts (15). A positive AI-enhanced ECG was associated with a 3.9- to 24-fold higher risk of new-onset HF compared with traditional risk models. Complementing these findings, a companion study validated a single-lead, wearable-compatible AI-enhanced ECG (15, 16), demonstrating that clinically meaningful predictions can be achieved using minimal signals collected by consumer devices. Together, these results indicate that AI-enhanced ECG has the potential to detect HF-related risk signatures well before conventional clinical recognition.

Prognostic applications are also emerging. Cho et al. developed the Quantitative Electrocardiographic, or QCG, score derived from printed ECGs, which predicted both in-hospital cardiac death and long-term mortality in patients with acute HF. Such tools may support frontline clinicians by identifying high-risk patients early during hospitalization (17).

Remote monitoring is another promising application. A recent feasibility study evaluated an AI-enabled smartwatch ECG system designed to predict 30-day rehospitalization in patients with established HF. This approach leverages daily ECG-based monitoring combined with AI algorithms to detect early signs of decompensation, enabling timely interventions. If validated, it could expand continuous monitoring and support earlier detection of clinical deterioration, with potential benefits in reducing readmissions and improving quality of life (18).

Interpretability also remains a key area of development. While some models still operate as black boxes, others now incorporate explainable AI methods that highlight the ECG features most predictive of HF, improving transparency and clinician trust (19). Seamless integration into electronic health records and consumer devices will be essential to realize the full clinical impact of AI-enhanced ECG (20).

### AI-enabled stethoscopes in primary care

Auscultation is one of the oldest diagnostic techniques in medicine, but its diagnostic accuracy is limited by interobserver variability. Integrating artificial intelligence into digital stethoscopes enables clinicians to obtain real-time, objective assessments of cardiac function, reducing subjectivity and potentially improving early detection.

A recent pragmatic randomized trial conducted in Nigeria showed that the use of an AI-enabled digital stethoscope doubled the detection of pregnancy-related cardiomyopathy compared with routine obstetric care (21). The device demonstrated excellent diagnostic performance, with an area under the curve of approximately 0.97–0.98 for detecting left ventricular ejection fraction below 50 percent, and it provided immediate, point-of-care results. These findings highlight the potential of AI-enhanced auscultation to improve early diagnosis in resource-limited settings where access to echocardiography is scarce (21).

In a multicenter study involving 2,960 adults across four U.S. health care networks, an AI-enabled digital stethoscope achieved an area under the receiver operating characteristic curve of 0.85 and a negative predictive value of 98% for detecting an ejection fraction ≤40%. These findings demonstrate its potential as a scalable and accessible screening tool in routine clinical care (10).

Efforts to scale implementation are underway through the Triple Cardiovascular Disease Detection with an Artificial Intelligence-Enabled Stethoscope (TRICORDER) trial, a pragmatic cluster-randomized study involving up to 200 primary care practices in the United Kingdom. The trial is designed to evaluate diagnostic performance for heart failure, atrial fibrillation, and valvular disease, as well as workflow feasibility, health economic outcomes, and factors influencing adoption. It represents one of the most comprehensive real-world evaluations of AI-enabled stethoscopes to date (22).

Time efficiency is another advantage. A recent study showed that an AI-enabled stethoscope could analyze heart sounds and provide a diagnostic output for left-sided valvular disease in approximately ninety-one seconds. Although this study was conducted in a hospital setting, the ability to generate actionable information within a single consultation illustrates the potential of these tools to shorten diagnostic pathways that traditionally require weeks through referral-based systems (23).

Importantly, AI-enabled stethoscopes may help reduce global disparities in cardiovascular care. In regions where access to echocardiography is limited or absent, these devices can extend diagnostic capacity to community clinics and rural health centers, offering a low-cost and portable alternative that empowers non-specialists and broadens access to early HF detection.

### System-level integration and economic considerations

**Figure 1:**
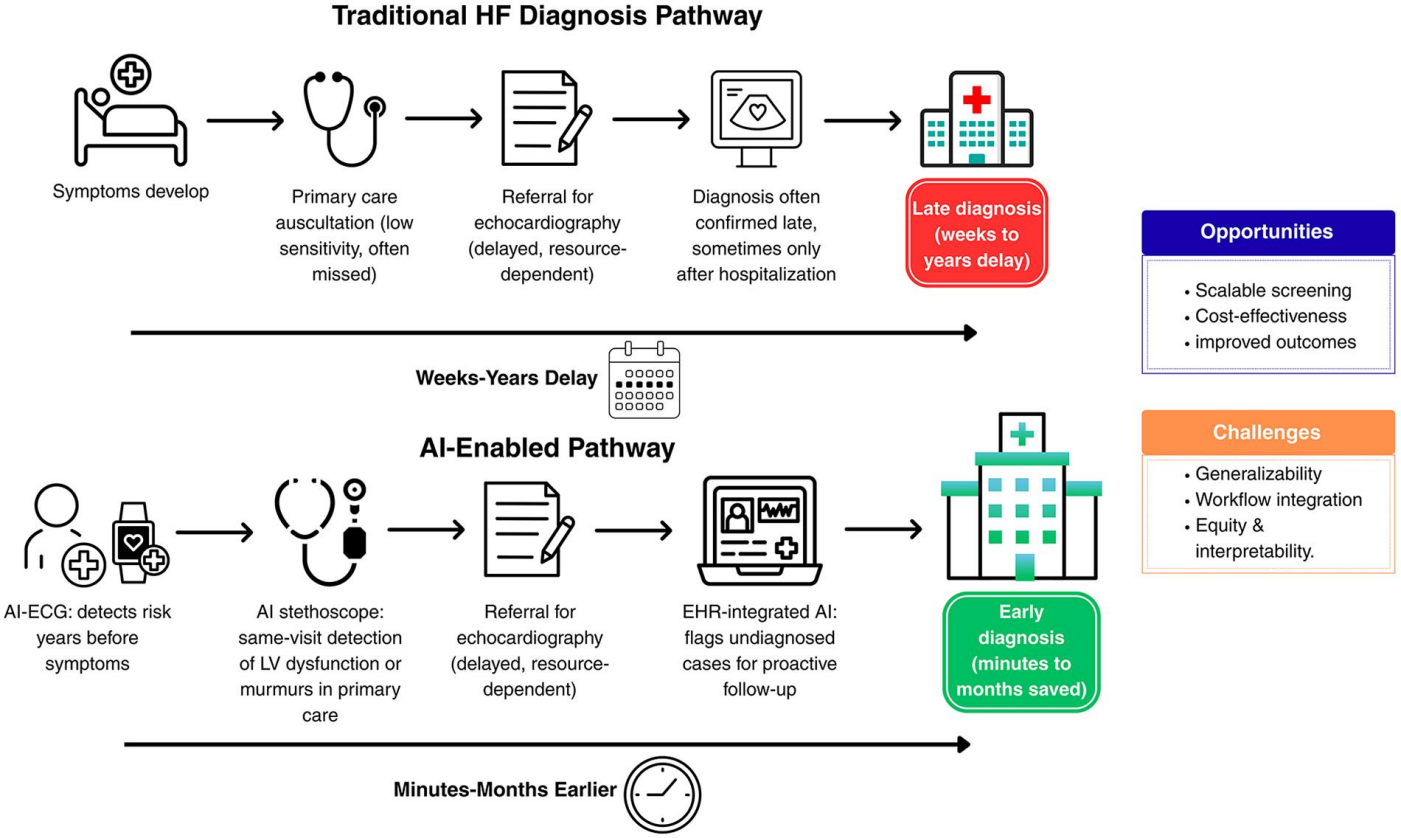
Traditional vs. AI-Enabled Pathways for Heart Failure Diagnosis. HF, heart failure; AI, artificial intelligence; ECG, electrocardiogram; Echo, echocardiography; LV, left ventricle; EHR, electronic health record. Original figure created by the authors.

The transition from device-level innovations to system-wide integration can be illustrated by contrasting the traditional diagnostic pathway with an AI-enabled approach (Figure 1). Conventional pathways often lead to delayed recognition because auscultation has limited sensitivity, and echocardiography is not always accessible. In contrast, AI-based tools offer earlier identification through ECG-driven risk stratification, same-visit AI-assisted auscultation, and proactive case detection from electronic health records. These differences highlight both the opportunities and the challenges of embedding AI into routine clinical practice.

For artificial intelligence to progress beyond pilot studies, it must be incorporated into existing health systems and demonstrate sustainable value at scale (24). Economic feasibility is a central consideration. A recent cost-effectiveness analysis of AI-enhanced ECG screening for asymptomatic left ventricular systolic dysfunction in outpatient settings reported that the strategy was cost-saving, with an incremental cost-effectiveness ratio of minus 7,439 United States dollars. With more than a 90 percent probability of cost-effectiveness under conventional willingness-to-pay thresholds, these findings provide strong support for reimbursement models that facilitate adoption in routine care (25).

Beyond device-specific applications, AI can strengthen system-wide detection strategies. For instance, algorithms applied to electronic health records have successfully identified patients with previously undiagnosed HF, achieving clinically meaningful accuracy (26). Such approaches demonstrate how AI can be integrated into routine workflows, enabling health systems to proactively identify individuals at risk who might otherwise remain unrecognized (24).

However, several challenges persist. Many algorithms have been trained on relatively homogeneous populations, raising concerns about their generalizability across diverse ethnic and geographic groups. Workflow integration also remains complex, as clinicians may hesitate to adopt new technologies unless they are seamlessly incorporated into practice and generate outputs that are interpretable and actionable. Advances in explainable AI, such as methods based on SHapley Additive exPlanations that highlight the variables most predictive of HF severity, represent meaningful progress toward improving transparency and clinician trust (27).

Despite these promising advances, several barriers to real-world deployment remain. AI models may experience performance drift over time as population characteristics and care pathways evolve, underscoring the need for continuous monitoring and recalibration. Most algorithms have been trained and validated on specific devices or health systems, raising concerns about generalizability when deployed across heterogeneous infrastructures. Data quality, incomplete EHR inputs, and variability in signal acquisition further challenge reliability. Finally, without clear clinical guidelines and implementation frameworks, clinicians may remain hesitant to incorporate AI outputs into decision-making. Addressing these system-level limitations is essential for safe and scalable adoption.

Equity is perhaps the most pressing issue. Without deliberate policies, AI innovations risk widening disparities by disproportionately benefiting well-resourced health systems. Evidence from large-scale evaluations has shown that algorithms trained on health care expenditures as a proxy for clinical need can inadvertently disadvantage minority populations, underestimating risk in groups with historically limited access to care. This concern is especially relevant for HF, which has a higher prevalence in lower-income populations that often lack access to echocardiography. Ensuring fairness in the design, validation, and implementation of AI tools must therefore remain a central priority (28).

## Discussion and call to action

Artificial intelligence for early HF detection is shifting from theoretical potential to practical clinical utility. AI-enhanced ECG provides scalable screening and prognostic refinement, and AI-enabled stethoscopes offer immediate point-of-care diagnostic support. These innovations are increasingly supported by prospective validation and evidence of cost effectiveness (8, 29). In contrast with earlier reviews, this manuscript highlights the combined impact of AI-enhanced ECG, AI-enabled auscultation, and system-level integration, emphasizing how these domains together may reshape the diagnostic pathway for HF.

The following steps are clear. Large, multicenter, pragmatic trials, such as TRICORDER, are essential for establishing external validity and determining the real-world impact of these tools on patient outcomes. Clinicians will require structured training not only in device use but also in interpretation, ensuring that AI complements rather than replaces clinical reasoning (30, 31). Regulatory bodies and professional societies should advance clear standards for validation, transparency, and integration of AI-based diagnostics into routine workflows. Payers and policymakers must also adapt reimbursement models that support adoption, particularly as evidence accumulates that AI-based HF screening can be cost-saving (32).

Equity must remain central. Without intentional strategies, AI-based tools may become concentrated in well-resourced health systems, leaving behind populations with the greatest unmet need. International agencies and national health authorities should prioritize pilot programs in low and middle-income countries, with a focus on sustainable community-level implementation. Transparent reporting of algorithm performance across diverse populations will be critical to building trust and avoiding the perpetuation of existing disparities (33, 34).

Regulatory momentum is another critical consideration. The European Union's AI Act and evolving guidance from the United States Food and Drug Administration on AI- and machine-learning-enabled medical devices represent early steps toward structured oversight (35, 36). These frameworks will influence how HF detection tools are evaluated, deployed, and monitored. Active engagement between the cardiology community and regulatory stakeholders will be essential to ensure that innovation progresses safely, effectively, and ethically (8).

In summary, AI should be viewed as a catalyst for earlier, more equitable, and more cost-effective HF detection. The field is now positioned to move beyond proof-of-concept models toward broad clinical implementation, supported by robust clinical trials, appropriate reimbursement, regulatory alignment, and commitment to equitable distribution.

## Conclusion

Artificial intelligence is rapidly transitioning from research settings to clinical cardiology. In heart failure, its most significant promise lies in earlier recognition: AI-enhanced ECGs can detect risk-related patterns before symptoms become apparent, and AI-enabled stethoscopes can compress traditional diagnostic delays into a single consultation. Supported by emerging evidence of cost-effectiveness, these tools have the potential to shift detection from late-stage crisis care to proactive, timely management.

The key challenge is no longer whether AI can detect HF, but how to implement it responsibly. Successful adoption will require rigorous multicenter validation, seamless integration into routine clinical workflows, clinician training, and equitable access across diverse health care systems. If these conditions are met, AI will not replace clinical judgment but will enhance it, helping transform HF detection into a faster, fairer, and more effective paradigm for patients worldwide.
